# Secretory High-Mobility Group Box 1 Protein Affects Regulatory T Cell Differentiation in Neuroblastoma Microenvironment* In Vitro*

**DOI:** 10.1155/2018/7946021

**Published:** 2018-12-16

**Authors:** Thitinee Vanichapol, Wararat Chiangjong, Jirawan Panachan, Usanarat Anurathapan, Somchai Chutipongtanate, Suradej Hongeng

**Affiliations:** ^1^Hematology and Oncology Division, Department of Pediatrics, Faculty of Medicine Ramathibodi Hospital, Mahidol University, Bangkok 10400, Thailand; ^2^Pediatric Translational Research Unit, Department of Pediatrics, Faculty of Medicine Ramathibodi Hospital, Mahidol University, Bangkok 10400, Thailand; ^3^Department of Cancer Biology, University of Cincinnati College of Medicine, Cincinnati, OH 45267, USA

## Abstract

Neuroblastoma (NB) is the most common extracranial tumor of childhood with poor prognosis in a high-risk group. An obstacle in the development of treatment for solid tumors is the immunosuppressive nature of the tumor microenvironment (TME). Regulatory T cells (Tregs) represent a T cell subset with specialized function in immune suppression and maintaining self-tolerance. Tregs resident within the tumor milieu is believed to play an important role in immune escape mechanisms. The role of the NB microenvironment in promoting Treg phenotype has never been elucidated. Herein, we demonstrated that the NB microenvironment promoted T cell activation and one NB cell line, SK-N-SH, manifested an ability to induce Treg differentiation. We identified tumor-derived HMGB1 as a potential protein responsible for Treg phenotype induction. By neutralizing HMGB1, Treg differentiation was abolished. Finally, we adopted a dataset of 498 pediatric NB via the NCBI GEO database, accession GSE49711, to validate clinical relevance of HMGB1 overexpression. Up to 11% of patients had HMGB1-overexpressed tumors. Moreover, this patient subpopulation showed higher risks of tumor progression, relapse, or death. Our findings emphasize the importance of immunological signature of tumor cells for appropriate therapeutic approach. Upregulation of secretory HMGB1 may contribute to suppression of antitumor immunity through induction of Tregs in the NB microenvironment.

## 1. Introduction

Neuroblastoma (NB) is the most common pediatric solid malignancy that has heterogeneity in clinical presentation. Patients with high-risk NB have a dismal prognosis of less than 40% at five-year survival rate despite intensive therapies [1]. In the past few decades, an approach for adverse prognosis NB patients has shifted toward immunotherapy, i.e., anti-GD2 monoclonal antibodies and chimeric antigen receptor (CAR) T cells. The latter is being tested in a number of clinical trials [2]. However, the efficacy of immunotherapeutic modalities for solid tumors, including NB can be impeded by the immunosuppressive nature of tumor microenvironment (TME) [3, 4]. In order to improve the potency of immunotherapeutic strategies for NB, a profound understanding of immunosuppressive TME exploited by cancer cells is crucial [3, 4].

Tregs represent a small population of T lymphocytes, normally account for 5-10% of CD4^+^ T cells [5], and are considered to be a key mediator in maintaining peripheral tolerance. Tregs are comprised of natural Tregs (nTegs), which develop in the thymus, and induced Tregs (iTregs), which are derived from naïve CD4^+^ T lymphocytes under the influence of tolerogenic conditions and various factors such as IL-10 and TGF-*β* [6]. Both subsets of Tregs are traditionally characterized by expression of the Forkhead Box P3 (Foxp3) transcription factor, which confers suppressive function, and CD25, an activation marker [7]. The difference is that, unlike nTregs, Foxp3 expression of iTregs is relatively unstable [8]. Tregs mediate inhibitory function through multiples mechanisms including secretion of immunosuppressive cytokines (e.g., IL-10 and TGF-*β*), expression of inhibitory receptors (e.g., CTLA-4, PD-1, and LAG-3), direct cytolysis, metabolic disruption of T effector cells, and induction of tolerogenic dendritic cells (DCs) [7, 9].

The role of Tregs in solid tumors (both intratumoral and peripheral Tregs) and their association with clinical outcomes have remained controversial. On one hand, infiltrating Tregs may play protective roles by controlling inflammation [10]. On the other hand, Tregs may promote tumor progression by suppressing tumor-specific immune responses within the TME [10]. Several studies have evaluated the presence of Tregs in cancer patients [11–17]. The frequency of Tregs is correlated with favorable prognosis in colorectal cancer [11] and head and neck squamous cell carcinoma [12]. In other types of cancer such as breast cancer [13], pancreatic cancer [14], and soft tissue sarcoma [15], high numbers of Tregs have been associated with poor clinical outcome. Although limited data of Tregs in NB patients are available, a few studies found that NB patients had higher Treg frequency [16, 17] and therapeutic targeting of Tregs can improve antitumor immunity against NB [18–21].

The TME is comprised of cancer cells reside in a specialized niche made up of stromal cells, the vascular system, extracellular matrix, soluble proteins, and infiltrating immune cells including Tregs [3, 4]. A complex array of interactions among cells within the TME is modeled by cancer cells for their own benefits and is facilitated by various growth factors, cytokines, and tumor-derived proteins, i.e., secretomes. The protein composition of cancer secretomes is dynamic as a consequence of genetic mutations and a variety of interactions with external triggers [22]. An analysis of proteins secreted by mouse neuroblastoma showed that these proteins were involved in various aspects of cancer progression including cell proliferation, apoptosis, angiogenesis, and cell adhesion [23]. Although the presence of particular T cell subsets such as CD8^+^ T cells or Tregs within tumor lesions has been used as a prognostic factor for decades, the impacts of NB-derived secreted proteins on Treg differentiation within the TME are largely unknown, given that characterization of NB secretomes will give some insights into new therapeutic targeting of Tregs in the NB TME.

In the present study, we demonstrated that a NB cell line SK-N-SH, but not SK-N-AS or SH-SY5Y, produced the permissive TME for Treg differentiation from peripheral blood mononuclear cells (PBMCs). Secretomic profiling, bioinformatics, and functional studies revealed that tumor cell-derived high-mobility group box 1 (HMGB1) protein was a major contributor of Treg differentiation within the NB TME.

## 2. Materials and Methods

### 2.1. Cell Culture

Three neuroblastoma cell lines, SK-N-SH (ATCC HTB-1), SK-N-AS (ATCC CRL-213), and SH-SY5Y (ATCC CRL-226), were purchased from the American Type Culture Collection (ATCC; Manassas, VA). SK-N-SH and SK-N-AS represented neuroblastoma cells with different genetic background. SH-SY5Y cells are a subline of the parental line SK-N-SH with some morphologically distinct phenotypes. All three are MYCN-nonamplified cell lines. Cells were grown in RPMI 1640 medium (Caisson Labs; Smithfield, UT) with 10% fetal bovine serum (FBS) (Gibco; Fisher Scientific, Waltham, MA) and 1% penicillin/streptomycin (Gibco).

Human peripheral blood mononuclear cells (PBMCs) were obtained from five healthy donors and were isolated by density gradient centrifugation using Ficoll-Paque (GE healthcare; Little Chalfont, UK). PBMCs were then cultured in RPMI 1640 medium with 10% fetal bovine serum (FBS), 100 U/mL IL-2 (PeproTech; Rocky Hill, NJ) and 1% penicillin/streptomycin. All cells were maintained at 37°C with 5% CO_2_. This study was approved by the Ethical Clearance Committee on Human Rights Related to Research Involving Human Subjects, Faculty of Medicine Ramathibodi Hospital, Mahidol University (protocol ID 10-60-21).

For coculture experiments, a total number of 10^5^ cells of PBMCs were cocultured with each NB cell line at a ratio of 1:3 (PBMC: NB) in complete RPMI 1640 medium with IL-2 10 U/mL in 24-well culture plate. Cultures were maintained at 37°C 5% CO_2_. On day 4, the suspension cells were collected and analyzed by flow cytometry. NB cells, which were adherent cells, remained attached to the wells and were excluded from the analysis. PBMC culture without NB cells was served as a blank control.

### 2.2. Flow Cytometric Analysis

The fluorochrome-coupled monoclonal antibodies (mAbs) used in this study were anti-CD4 phycoerythrin (PE), anti-CD25 Phycoerythrin-Cyanin 7 (PE-Cy7) and anti-FoxP3 Fluorescein Isothiocyanate (FITC) (#MHCD0404, #25-0259-41, #11-4776-42; ThermoFisher; Florence, KY). The analysis of Treg markers was carried out on day 4 after coculture. Briefly, 10^5^ cells of PBMCs were first stained with anti-CD4-PE and anti-CD25-PE-Cy7 for 30 min at 4°C and washed 2 times in Dulbecco's phosphate buffered saline (DPBS) (GE healthcare) followed by intracellular staining using fixation and permeabilization kit (ThermoFisher). After washing in permeabilization buffer, cells were stained with FoxP3-FITC antibody for another 30 min at 4°C and were then analyzed by BD FACSVerse flow cytometry with BD FACSuite software (BD Bioscience; San Jose, CA). Tregs were identified as cells with CD4^+^CD25^+^FoxP3^+^ using sequential gating strategy.

### 2.3. Secretome Preparation

For collection of the culture media, SK-N-SH and SK-N-AS cells were plated in T-75 and cultured in complete RPMI 1640 medium until at 80% confluent. Cells were washed with PBS 4 times to remove serum protein contaminants and then cells were cultured in serum-free RPMI 1640 medium for 24h. Thereafter, the culture supernatants were collected and centrifuged at 1000g for 5 min to remove pellets and cell debris. Secretory proteins in the culture supernatants (20 mL per sample) were isolated and concentrated by ultrafiltration using Microsep™ Advance Centrifugal Devices (3 kDa cut-off) (PALL, Port Washington, NY, USA). The 2D Clean-Up Kit (GE healthcare) was used according to the manufacturer's instructions to remove waste products and contaminants that might interfere with further proteomic analysis. The protein pellet obtained from the 2D Clean-Up kit was resuspended in a lysis buffer containing 7M urea, 2M thiourea, 4% CHAPS, 40mM Tris, 120mM dithiothreitol (DTT), 2% ampholyte pH 3-10, and 1% protease inhibitor mix. Protein estimation was performed by Bradford assay. The secretome protein samples were stored at -80°C until used.

### 2.4. Two-Dimensional Gel Electrophoresis (2DE)

Secretome samples (80 *μ*g proteins) were mixed with a rehydration buffer (7 M urea, 2M thiourea, 2% CHAPS, 120mM DTT, 2% ampholyte pH 3-10, and bromophenol blue) and rehydrated into a 7 cm nonlinear immobilized pH gradient (IPG) strips of pH range 3–10 (GE healthcare) for 16-18 hours at room temperature. The first dimension separation (or isoelectric focusing) was performed by the Ettan IPGphor III IEF System (GE healthcare) at 20°C in a stepwise voltage increase to reach 8,333 Vhrs with the limited of 50 *μ*A/strip. After isoelectric focusing, the strips were equilibrated in the first equilibration buffer (6 M Urea, 112 mM Tris-HCl (pH 8.8), 30% glycerol, 4% SDS, and 130 mM DTT) for 15 min, followed by the second equilibration buffer containing the same compositions with 135 mM iodoacetamide (IAA) instead of DTT for 15 min. The second dimensional analysis was performed on 12.5% SDS-PAGE using SE260 mini-Vertical Electrophoresis Unit at 150 V for approximately 2 h. Staining of 2DE gels was performed using Coomassie Colloidal Blue G-250 staining.

### 2.5. Spot Quantification

Gels were scanned by ImageScanner III (GE Healthcare) and the images (n = 5 secretome samples per cell line) were analyzed using Image master 2D platinum software version 7.0 (GE healthcare). Normalization of each protein spot was carried out in relation to the total protein spot intensity in the same gel image. Analysis of the expression level of the protein spots was performed by Student's t-test. The protein spots that passed the threshold of fold-change >3 and* p*-value of <0.05 were considered significant and were subjected to protein identification by mass spectrometry.

### 2.6. Mass Spectrometry

Protein spots of interest were excised from the 2DE gels, washed with deionized (DI) water, destained using 50 mM NH_4_HCO_3_ in 50% acetonitrile (ACN) at 37°C for 15 min, and dried in a SpeedVac concentrator. The gel pieces were reduced by incubating in 10 mM DTT/100 mM NH_4_HCO_3_ at 56°C for 30 min. The reduced gel pieces were then washed with ACN and then dried with SpeedVac concentrator and were alkylated with 55 mM IAA/100 mM ammonium bicarbonate at room temperature for 30 min in the dark. The alkylated gel piece was washed with ACN and all liquid was removed using SpeedVac concentrator. The proteins were then digested by incubating 18 h at 37°C with trypsin (Promega Corporation, Madison, WI, USA). The trypsin reaction was stopped by adding 10 *μ*l of 5% trifluoroacetic acid (TFA)/ACN (ratio 1:2). The gel piece in solution was vigorously vortexed for 1 min before collecting the protein mixtures into a new collection tube and then dried by a SpeedVac.

The dried peptides were resuspended with 2 *μ*l of 0.1%TFA and spotted on MTP 384 target plate ground steel BC (Bruker Daltonik GmbH, Breman, Germany) and mixed with 2 *μ*l of 2.5 mg/ml alpha-cyano-4-hydroxycinnamic acid matrix substance in 80%ACN/0.1%TFA. After drying peptide spot solution, the target plate was operated on Ultraflex II MALDI-TOF/TOF (Bruker) mass spectrometer. The parent ions in each sample were detected at positive refractor mode with m/z 700-3500 Da, frequency 2000 Hz, 2.3 mV analog offset. Parent ions with signal to noise ratio more than 3 were further fragmented to transition ions in LIFT mode. Mass of parent ions and transition ions were combined into one.data file and search via Mascot server by using Biotool software (Bruker). Mascot search parameters included carbamidomethylation at cysteine residue for fixed modification, oxidation at methionine for variable modification, monoisotopic ion, ±200 ppm for peptide tolerance, ±0.5 Da for fragment ion tolerance, Swiss-Prot database, Homo sapiens taxonomy, 1+ charge state, and trypsin digestion with 1 missed cleavage allowed. Identified proteins were provided ions scores (>28) more than identity threshold and contained at least one significant peptide.

### 2.7. Western Blot

Secretome samples (20 *μ*g protein) were resolved in 12.5%SDS-PAGE gel and proteins were electrotransferred onto a nitrocellulose membrane (Merck Millipore, Burlington, MA). After blocking with 5% skim milk, the membrane was incubated with primary antibody, anti-HMGB1 (1:1000 dilution; Biolegend, San Diego, CA,) overnight at 4°C. After washing, the membranes were incubated with secondary antibody, goat anti-mouse immunoglobulins/HRP (1:2000 dilution; Dako, Santa Clara, CA) for 1 h. The membrane was then incubated with enhanced chemiluminescence reagent (GE Healthcare) and the immunoreactive bands were visualized by X-ray film exposure. Anti-GAPDH (1:5000 dilution; Abcam, Cambridge, UK) was used as a loading control with the same secondary antibody (1:10000 dilution; Dako).

### 2.8. Neutralization Assay

To validate a functional role of HMGB1 in Treg differentiation during the coculture of PBMCs and SK-N-SH cells, varied concentrations of anti-HMGB1 antibody (1 *μ*g/mL, 2 *μ*g/mL, or 3 *μ*g/mL) and IgG isotype control (1 *μ*g/mL) (Dako) were added to the coculture experiment as aforementioned. The suspended cells were collected and analyzed by flow cytometry on day 4.

To confirm that secreted HMGB1 in the secretomes, but not factors associated with direct cell-cell contact, play roles in Treg differentiation, the culture media containing SK-N-SH secretomes with the supplements of 10% FBS and IL-2 10U/mL was used to treat 10^5^ cells of PBMCs in the presence or absence of anti-HMGB1 antibody (1 *μ*g/mL, 2 *μ*g/mL, or 3 *μ*g/mL) or IgG isotype control (1 *μ*g/mL). On day 4, the treated cells were analyzed by flow cytometry. PBMCs cultured in complete RPMI 1640 medium with IL-2 10U/mL was used as a negative control. This experiment was performed in 3 biological replicates.

### 2.9. Statistical and Data Analysis

Data analysis was performed by Excel and R package MetaboAnalystR (https://www.metaboanalyst.ca) [24]. The results were expressed as mean ± SD. Statistical analysis was performed by t-test or ANOVA with Tukey HSD post hoc as appropriate. All experiments were carried out as 5 biological replicates unless otherwise stated.* P*-value < 0.05 was considered as statistically significant. A self-organized heatmap was based on Euclidean distance and average linkage. Functional annotation of significant proteins was performed by STRING (https://www.string-db.org) [25], Panther (http://www.pantherdb.org) [26], and David functional annotation (https://david.ncifcrf.gov) [27].

The Gene Expression Omnibus (GEO) (https://www.ncbi.nlm.nih.gov/geo) [28], accession GSE49711, was accessed to adopt HMGB1 mRNA expression in tumors (from RNA-seq) and clinical data of 498 patients previously reported as part of the MicroArray Quality Control-III/Sequencing Quality Control (MAQC-III/SEQC) study [29]. HMGB1 mRNA upregulation and downregulation were defined as the z-score of >1.25 and <-1.25, respectively. Associations between HMGB1 mRNA alterations and clinical outcomes (i.e., the occurrence of events and death) were determined by logistic regression and presented as the adjusted odds ratio (OR) with 95% confidence interval (CI) using R package “epiDisplay” (available via http://www.cran.r-project.org). MYCN amplification, a strong prognostic factor of neuroblastoma, was used to adjust the influence of HMGB1 mRNA alterations on clinical outcomes. P-value <0.05 was considered as statistical significance.

## 3. Results

### 3.1. Coculture of PBMCs with NB Cell Lines Showed Phenotypic Differentiation of CD4^+^T Cell Subsets

**Figure 1:**
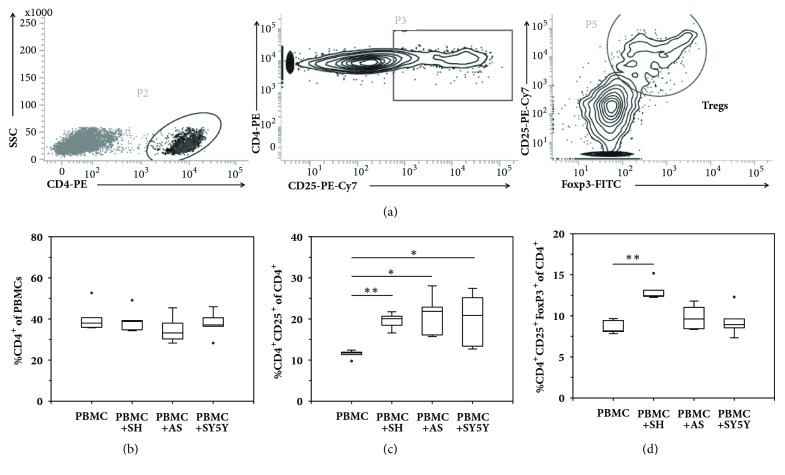
The expression of phenotypic Treg markers in coculture experiments by flow cytometry. PBMCs obtained from 5 healthy donors were cultured in the absence (PBMC) or presence of SK-N-SH (PBMC+SH), SK-N-AS (PBMC+AS), and SH-SY5Y (PBMC+SY5Y) for 4 days prior to flow cytometric analysis. (a) The sequential gating strategy of Treg enumeration. (b) The percentage of CD4^+^T cells of PBMCs. (c) The percentage of CD4^+^CD25^+^activated T cells of total CD4^+^T cells. (d) The percentage of CD4^+^CD25^+^Foxp3^+^Treg cell of total CD4^+^T cells. The results showed that the PBMC+SH coculture condition, but not those of PBMC+AS or PBMC+SY5Y, had significant increase in the Treg frequency as compared to the control condition (PBMC), suggesting that a particular microenvironment of SK-N-SH cells mediated Treg differentiation. Values represent mean±SD of 5 independent experiments (^∗^*p*<0.05; ^∗∗^*p*<0.001).

To investigate whether NB cell lines could generate a permissive microenvironment that mediated T cell phenotypic changes toward Treg differentiation, three NB cell lines, SK-N-SH, SK-N-AS, and SH-SY5Y were cocultured with PBMCs (n=5 healthy individuals) for 4 days and then analyzed for Treg markers by flow cytometry. The sequential gating strategy for Treg enumeration is presented in Figure 1(a). At baseline, PBMCs culture alone showed 40.5±7.1% of CD4^+^T lymphocytes (Figure 1(b)), 11.4±1.0% of activated CD4^+^CD25^+^T cells (Figure 1(c)), and 8.6±0.9% of CD4^+^CD25^+^Foxp3^+^Tregs (Figure 1(d)), respectively. After being cocultured with three NB cell lines, the percentage of CD4^+^T cells remained unchanged (Figure 1(b)); however, there were significant increases in the percentage of CD4^+^CD25^+^T lymphocytes (up to 20%) in all cocultured conditions (Figure 1(c)). Interestingly, only PBMCs coculture with SK-N-SH cells showed significantly higher number of CD4^+^CD25^+^Foxp3^+^Tregs as compared to the PBMCs culture alone (13.07±1.22% versus 8.64±0.85%,* p*<0.001) (Figure 1(d)), suggesting that some particular factors resided in the TME of SK-N-SH cells could augment Treg differentiation.

### 3.2. Analysis of Protein Differential Expression in SK-N-SH versus SK-N-AS Secretomes

**Figure 2:**
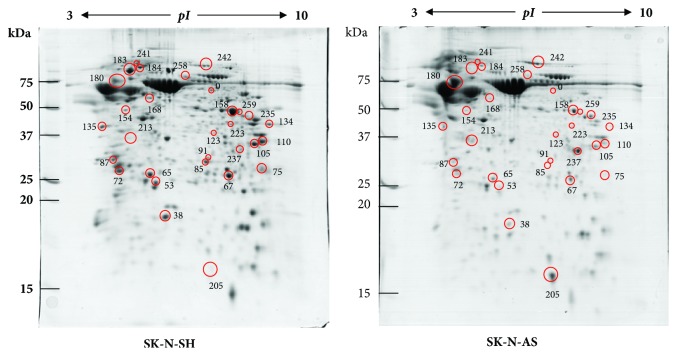
Representative 2DE gel images showing differentially expressed proteins in SK-N-SH versus SK-N-AS secretomes (n=5 per group). Thirty significantly altered protein spots were labeled by red circles with spot ID in which their corresponding data were reported in Table 1 and Supplementary Table 1.

It is hypothesized that Treg differentiation was mediated by unique molecules that only presented in SK-N-SH-derived secretomes. To elucidate this hypothesis, a comparison of secretomic profiles of SK-N-SH versus SK-N-AS was performed by proteomic analysis. We chose SK-N-AS over SH-SY5Y (a subline of SK-N-SH) since this cell line has a different genetic abnormality background to SK-N-SH. Representative gel images of the SK-N-SH and SK-N-AS secretomic profiles (5-independent samples/group) are shown in Figure 2. A total of 30 significant protein spots were detected at the threshold of fold-change of >3 and* p-*value of <0.05 between groups (Figure 2) and were analyzed by LC-MS/MS for 29 unique protein identities (Table 1 and Supplementary Table 1).

### 3.3. Protein Bioinformatics Revealed Secretory HMGB1 May Involve in Treg Differentiation

**Figure 3:**
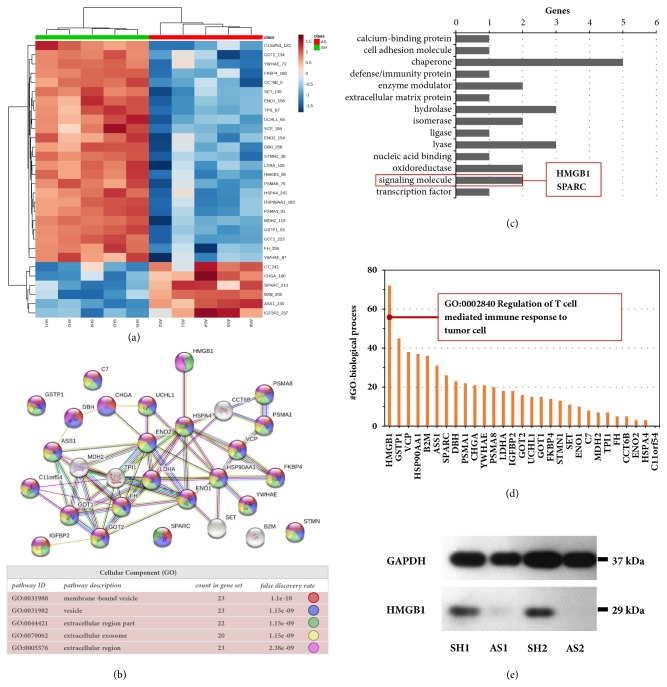
Protein bioinformatics and validation of HMGB1 expression. (a) A self-organized heatmap shows the relative expressional data of 29 significantly altered proteins (labeled by gene name with spot ID) in the secretomes of SK-N-SH (SH) versus SH-N-AS (AS) (n=5 per group). (b) STRING protein network with GO-cellular component analysis showed that 24 out of 29 proteins (colored nodes) were annotated as proteins in extracellular compartments (details in Supplementary Table 2). Gray nodes represented nonsecretory proteins. (c) Panther protein classification suggested that only 2 altered proteins functioned as the signaling molecules, in which one of them was HMGB1 (details in Supplementary Table 3). (d) David annotation of GO-term biological process showed that HMGB1 play multifunctional roles, one of which may involve in the regulation of T cell response to tumor cells (details in Supplementary Table 4). (e) HMGB1 levels in the SK-N-SH (SH) and SK-N-AS (AS) secretomes were validated by Western blot analysis in duplicate. GAPDH served as a loading control.

The significant proteins from proteomic analysis were then annotated by bioinformatic tools to pinpoint the unique proteins that potentially mediated Treg differentiation in the NB TME.  Figure 3(a) showed the relative abundance of 29 significantly altered proteins between SK-N-SH and SK-N-AS secretomes in a self-organized heatmap, in which two major clusters of 23 upregulations and 6 downregulations were detected. We considered that Treg-inducible proteins in SK-N-SH-derived secretomes should belong to the upregulation cluster. STRING protein network with GO-term cellular component analysis showed that 24 proteins were enriched in extracellular compartments, representing cell secretory products (Figure 3(b) and Supplementary Table 2). PANTHER protein classification showed that a large proportion of the identified proteins were chaperones, whereas there were 2 proteins that belong to the class of signaling molecule, SPARC and HMGB1 (Figure 3(c) and Supplementary Table 3). David functional annotation showed that, of all 29 significant proteins, only HMGB1 may involve in the regulation of T cell response to tumor cells (GO:0002840) (Figure 3(d) and Supplementary Table 4). Based on bioinformatic analyses, a literature review focusing on HMGB1 was performed. Since previous studies supporting its potential effects on Treg immunomodulation [30, 31], we chose HMGB1 as a candidate protein for further studies. Before functional validation, Western immunoblot analysis was used to confirm HMGB1 protein expression in secretomic samples. As expected, SK-N-SH-derived secretomes contained a higher level of HMGB1 as compared to those of SK-N-AS (Figure 3(e)).

### 3.4. Neutralization of Secretory HMGB1 Suppressed Treg Differentiation in the NB TME 

**Figure 4:**
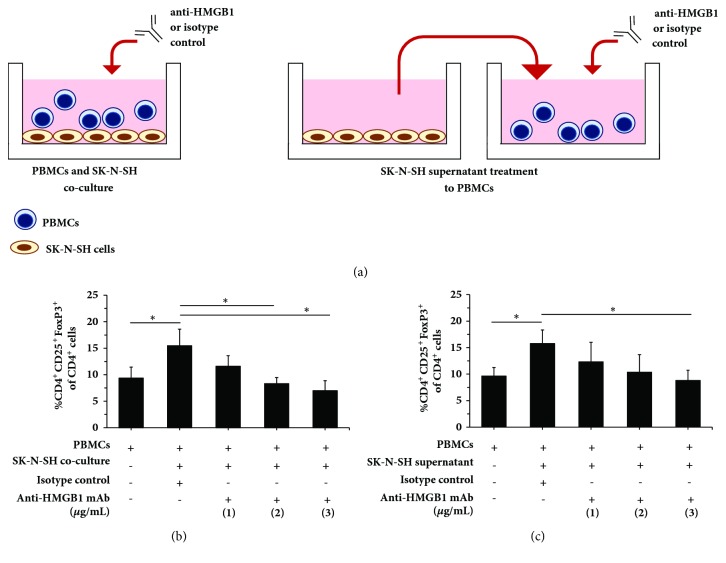
Neutralization by anti-HMGB1 inhibits Treg differentiation in a dose-dependent fashion. (a) Schematic diagram represents HMGB1 neutralization in the coculture (left panel) and the supernatant treatment (right panel) models. (b) The frequency of CD4^+^CD25^+^Foxp3^+^Tregs of CD4^+^T cells after PBMCs and SK-N-SH cell coculture in the presence of anti-HMGB1 mAb (1, 2, or 3 *μ*g/mL). (c) The frequency of CD4^+^CD25^+^Foxp3^+^Tregs of CD4^+^T cells after the SK-N-SH supernatant treatment to PBMCs in the presence of anti-HMGB1 mAb (1, 2, or 3 *μ*g/mL). Cells were collected on day 4 for Treg enumeration. Baseline Treg levels were measured in PBMCs culture using complete media at day 4. Goat IgG (1 *μ*g/mL) was served as the isotype control. PBMCs were obtained from 3 healthy individuals. Values represent mean±SD of 3 independent experiments (^∗^*p*<0.05).

To elucidate whether HMGB1 in the SK-N-SH secretomes play a major role in Treg differentiation, neutralization studies using anti-HMGB1 mAb were performed in the coculture and supernatant treatment models (Figure 4(a)). Consistent with previous data (Figure 1(d)), CD4^+^CD25^+^Foxp3^+^Treg differentiation was observed after PBMCs coculture with SK-N-SH NB cells for 4 days (Figure 4(a), left panel, and Figure 4(b)). Interestingly, addition of anti-HMGB1 mAb to the coculture suppressed Treg differentiation in a dose-dependent manner (Figure 4(b)). This result suggested that HMGB1 was an important factor for Treg differentiation in NB TME constituting both cancer cells and their secretomes.

To further validate that only secretory HMGB1 but not costimulatory factors of cell-to-cell contact was required for Treg differentiation, PBMCs were then solely treated with SK-N-SH culture supernatant (Figure 4(a), right panel) and subsequently subjected to the neutralization assay. As expected, SK-N-SH supernatant treatment resulted in the increased CD4^+^CD25^+^Foxp3^+^Treg levels (Figure 4(c)) as similar as those of the coculture experiment (Figure 4(b)). Again, anti-HMGB1 mAb neutralization could suppress Treg differentiation in dose-dependency (Figure 4(c)). This finding demonstrated that secretory HMGB1 play a central role of Treg differentiation in NB TME.

### 3.5. Integrated Analysis Showed HMGB1 Overexpression in Neuroblastoma Tumors Associated with Poor Clinical Outcomes

From a clinical standpoint, it is very interesting to address whether or not HMGB1 is overexpressed in patient tumors, and if so, that correlates at what extent to clinical outcomes. To gain some insight into the clinical relevance of HMGB1, we accessed the NCBI GEO database, accession GSE49711 (the MAQC-III/SEQC study) [28, 29], to adopt clinical and gene expression data of 498 pediatric neuroblastomas, focusing on determining the effects of HMGB1 expression in patient tumors associated with clinical outcomes (details in the Materials and Methods).  Table 2 showed patient characteristics and numbers of patients with HMGB1 mRNA alterations in tumors. Overall, the MAQC-III/SEQC cohort covered the entire spectrum of the disease [29]. Patients with HMGB1-overexpressed tumors presented up to 11% of all cases (55/498) in this cohort.

**Figure 5:**
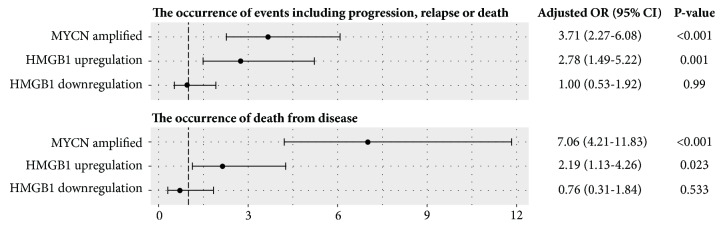
Association between HMGB1 expression in tumors and clinical outcomes in 498 neuroblastoma patients. Forest plot demonstrated that HMGB1 mRNA upregulation was an independent risk of poor clinical outcomes including disease progression, relapse, or death. MYCN amplification, a strong prognostic factor of neuroblastoma, was included in the model to adjust the contributed risk of HMGB1 mRNA alterations. Upregulation and downregulation of HMGB1 mRNA were defined as the z-score >1.25 and <-1.25, respectively. Data were made available in Supplementary Table 5.

Association analysis was performed to elucidate the effects of HMGB1 overexpression in NB tumors on clinical outcomes. Interestingly, the patients with HMGB1 mRNA upregulation in tumors showed significant associations with the occurrence of events including progression, relapse or death (adjusted OR of 2.78, 95% CI of 1.49-5.22;* p*=0.001), and the occurrence of death from disease (adjusted OR of 2.19, 95% CI of 1.13-4.26;* p*=0.023), whereas the patients with HMGB1 mRNA downregulation in tumors had no association to unfavorable outcomes (Figure 5). Direct evidence of clinical associations, together with the findings of Treg differentiation due to high HMGB1 levels in NB TME* in vitro*, supported future investigations which aim to mitigate HMGB1 overexpression in tumors and/or to neutralize secretory HMGB1 in NB TME.

## 4. Discussion

During the past few decades, a large body of evidence has uncovered a pivotal role of the TME in solid tumors in escaping host immunity. However, limited data are available on the significance of circulating and intratumoral Tregs in NB patients. Morandi et al. (2015) reported higher percentage of circulating Tregs in metastatic patients than healthy controls [17]. The finding is in accordance with those demonstrated by Tilak et al. (2014) [16]. Despite ambiguous data on the Treg frequency and the clinical outcome, depletion of these immunosuppressive T cells seems to be beneficial in cancer treatment [20, 32–34]. Transient depletion of either CD4^+^ or CD25^+^ T cells has been shown to enhance immunotherapy and antitumor immunity in murine NB models [19, 20, 35, 36].

In the present study, we sought to gain a better understanding of NB-derived secretomes involved in promoting Treg immunophenotype. A study by Carlson et al. (2013) compared CD25 surface expression of peripheral blood lymphocytes (PBLs) and tumor-associated lymphocytes (TALs) from NB patients and found a larger proportion of T cells expressing CD25 in autologous TALs indicating that the NB microenvironment can promote T cell activation [37]. The same group also reported CD25 upregulation when PBLs were cultured in the presence of autologous tumor cells. Consistent with the previous study, the coculture experiments in our study demonstrated that CD25 expression was upregulated when PBMCs were cultured with NB cells (Figure 1(c)); thus the TME is permissive for T cell activation. However, Carlson et al. (2013) did not find Foxp3 upregulation in TALs as compared to PBLs [37]. In contrast, we found that one NB cell line, SK-N-SH, exhibited an ability to induce Treg differentiation, while the other two cell lines, SK-N-AS and SH-SY5Y, did not share this property. One possible explanation for this discrepancy may be attributable to a wide diversity of genetic mutations of NB tumors resulting in different immunological signature. The influence of the TME on Treg induction has been reported in other type of solid tumors such as hepatocellular carcinoma [38] and ovarian [39]. For example, coculture CD8^+^ cells with ovarian cancer cell line can induce CD8^+^ Tregs that can inhibit naïve CD4^+^ T cell proliferation [39].

Tumor secreted proteins play important roles in cell-to-cell communication and modulating host immune response in the TME niche. Up to date, several soluble factors which confer immunomodulatory properties have been identified. Based on the previous studies [30, 31], HMGB1 is a potential candidate as an immune suppressive factor in our study. HMGB1 is a member of the danger associated molecular patterns (DAMPs) family that has divergent biological functions including inflammation, autophagy/apoptosis, promotes tumor cell survival, and mediates immune responses. The protein can engage with several receptors including advanced glycation end products (RAGE) and Toll-like receptors (TLRs) [40]. Extracellular HMGB1 is released by both cancer and infiltrating immune cells within the inflammatory TME. It can be found in the nucleus, the cytoplasm, on the cell surface, and the extracellular environment [41]. HMGB1 has been shown to play a role in both immunosuppressive and immune-activation properties [40]. As an immune-activating factor, HMGB1 released from necrotic tumor cells binds to Toll-like receptor 4 (TLR4) expressed by dendritic cells (DCs) to promote antigen presentation and also activates macrophage TNF release and thus activates antitumor immunity [42, 43]. Conversely, tumor-derived HMGB1 may favor tumor progression and suppress antitumor immunity by promoting IL-10 production in Tregs through RAGE [30, 31]. HMGB1 is expressed in various cancers and is associated with cancer progression [44]. The protein is associated with tumor growth and chemoresistance in NB [45, 46]. HMGB1 overexpression using a lentivirus in SH-SY5Y cells was found to promote cell growth and migratory ability [46]. Another major role of HMGB1 is to function as a mediator of autophagy, which contributes to chemoresistance [41, 46, 47]. HMGB1 expression in cancer cells was upregulated following exposure to anticancer agents resulting in translocation of the protein to the cytoplasm [46, 47]. Cytosolic HMGB1 competes with Bcl-2 in binding with Beclin 1 leading to the formation of autophagosomes [47, 48]. Nonetheless, the role of HMGB1 in Tregs is still controversial. Preincubation of Tregs with HMGB1 has been found to reduce their inhibitory function and IL-10 secretion [49], while the other group demonstrated increased suppressive function and prolong survival of Tregs after HMGB1 stimulation [31].

Despite the findings from previous studies, the role of NB-derived HMGB1 in promoting Treg differentiation has never been elucidated. The observations in this study suggested that NB-derived secretory HMGB1 enhance Treg differentiation by upregulating Foxp3, the transcription factor that dominantly controls Treg suppressive capacity [7], in activated CD4^+^CD25^+^T cells (Figures 1(b) and 1(c)). Inhibition of HMGB1 by the specific antibody significantly reduced the number of CD4^+^CD25^+^Foxp3^+^Tregs in the dose-dependent manner (Figures 4(b) and 4(c)). By the fact that up to 11% of NB patients had HMGB1 overexpression in tumors (Table 2) and this was associated with the increased risk of adverse events including tumor progression, relapse, or death (Figure 5), antitumor immunity mediated by HMGB1-induced Treg differentiation may be responsible for unfavorable outcomes. This finding has a translational potential, where HMGB1 in the NB TME is investigated as a therapeutic targeting of Tregs to improve antitumor immunity against NB in the future.

There are a number of limitations in our study. We did not examine the suppressive function of Tregs in the presence of NB cells. In addition, the mechanism of HMGB1-mediated Treg differentiation in our study including receptors and downstream signaling pathways remains to be elucidated. The limitation in the nature of the* in vitro* system should also be taken into account as we neglected the interaction between other cell types and tumor cells present in the* in vivo* environment. Furthermore, we did not examine the function of other significant proteins in SK-N-SH secretomes. Besides these limitations, our data showed that HMGB1 secreting NB cells could induce Treg differentiation* in vitro *and thus may serve as a potential therapeutic target in cancer immunotherapy.

## 5. Conclusions

In conclusion, our data demonstrate that the NB microenvironment is permissive of T lymphocyte activation and HMGB1 secreting NB cells can promote Treg differentiation. We propose HMGB1 as the major contributor of Treg differentiation in the NB TME. Further studies focusing on HMGB1-mediated Treg differentiation are warranted to mitigate immunosuppressive microenvironment which eventually improve the efficacy of NB immunotherapy.

## Figures and Tables

**Table 1:** Summary of differentially expressed proteins between SK-N-SH versus SK-N-AS secretomes (detailed information regarding peptide sequences and raw spot intensities in Supplementary Table 1).

**Spot ID**	**Gene name**	**Protein name**	**SwissProt ID**	**Scores**	%** Cov**	**No. of distinct peptides**	**p*I***	**MW (kDa)**	**Spot intensity** **Mean±SEM**		**Fold change** ^∗^	***p*-value**
									**SK-N-SH**	**SK-N-AS**		
*Protein spots increased in SK-N-SH secretome*												
0	CCT6B	T-complex protein 1 subunit zeta-2	Q92526	81	2	1	6.85	58.35	0.245 ± 0.015	0.079 ± 0.011	3.10	1.59E-05
38	STMN1	Stathmin	P16949	154	9	2	5.76	17.29	0.580 ± 0.028	0.130 ± 0.022	4.43	1.43E-06
53	GSTP1	Glutathione S-transferase	P09211	46	5	1	5.43	23.57	0.418 ± 0.027	0.095 ± 0.011	4.41	3.60E-06
65	UCHL1	Ubiquitin carboxyl-terminal hydrolase isozyme L1	P09936	237	21	3	5.33	25.15	0.519 ± 0.062	0.145 ± 0.016	3.58	3.71E-04
67	TPI1	Triosephosphate isomerase	P60174	214	12	2	5.65	31.05	0.992 ± 0.124	0.202 ± 0.020	4.92	2.28E-04
72	YWHAE	14-3-3 protein epsilon	P62258	34	10	3	4.63	29.32	0.678 ± 0.024	0.168 ± 0.043	4.04	7.08E-06
75	PSMA8	Proteasome subunit alpha type-7-like	Q8TAA3	60	5	1	9.07	28.68	0.707 ± 0.081	0.119 ± 0.032	5.94	1.41E-04
85	HMGB1	High mobility group protein B1	P09429	217	13	2	5.62	25.04	0.354 ± 0.058	0.028 ± 0.010	12.83	5.74E-04
87	YWHAE	14-3-3 protein epsilon	P62258	158	11	3	4.63	29.32	0.279 ± 0.033	0.083 ± 0.017	3.37	7.60E-04
91	PSMA1	Proteasome subunit alpha type-1	P25786	227	17	3	6.15	29.82	0.119 ± 0.008	0.035 ± 0.004	3.43	1.67E-05
105	LDHA	L-lactate dehydrogenase A	P00338	115	11	3	8.44	36.95	0.572 ± 0.049	0.178 ± 0.028	3.22	1.17E-04
110	MDH2	Malate dehydrogenase, mitochondrial	P40926	375	17	4	8.92	35.90	0.894 ± 0.033	0.217 ± 0.031	4.11	4.07E-07
123	C11orf54	Ester hydrolase C11orf54	Q9H0W9	36	2	1	6.23	35.60	0.146 ± 0.014	0.044 ± 0.006	3.31	1.96E-04
134	GOT2	Aspartate aminotransferase, mitochondrial	P00505	149	6	2	9.14	47.88	0.282 ± 0.016	0.074 ± 0.019	3.82	3.36E-05
135	SET	SET protein	Q01105	94	7	2	4.23	33.47	0.407 ± 0.021	0.132 ± 0.017	3.07	7.65E-06
154	ENO2	Gamma-enolase	P09104	371	12	4	4.91	47.58	0.132 ± 0.014	0.041 ± 0.008	3.23	5.46E-04
158	ENO1	Alpha-enolase	P06733	243	11	3	7.01	47.48	1.390 ± 0.148	0.374 ± 0.039	3.72	1.62E-04
168	FKBP4	Peptidyl-prolyl cis-trans isomerase	Q02790	189	6	2	5.35	52.05	0.206 ± 0.009	0.051 ± 0.010	4.02	3.51E-06
183	HSP90AA1	Heat shock protein HSP 90-alpha	P07900	393	9	5	4.94	85.00	0.933 ± 0.125	0.101 ± 0.030	9.28	1.89E-04
184	VCP	Transitional endoplasmic reticulum ATPase	P55072	128	2	1	5.14	89.95	0.631 ± 0.114	0.131 ± 0.012	4.79	2.45E-03
223	GOT1	Aspartate aminotransferase, cytoplasmic	P17174	340	22	5	6.52	46.44	0.163 ± 0.009	0.041 ± 0.005	4.00	3.83E-06
241	HSPA4	Heat shock 70 kDa protein 4	P34932	185	3	2	5.11	95.12	0.114 ± 0.009	0.036 ± 0.007	3.14	1.70E-04
258	DBH	Dopamine beta-hydroxylase	P09172	191	5	2	5.97	69.87	0.230 ± 0.021	0.066 ± 0.012	3.48	1.37E-04
259	FH	Fumarate hydratase	P07954	296	8	2	8.85	54.77	0.123 ± 0.012	0.029 ± 0.056	4.15	9.83E-05
*Protein spots decreased in SK-N-SH secretome*												
180	CHGA	Chromogranin-A	P10645	215	7	2	4.58	50.82	0.629 ± 0.091	2.092 ± 0.366	0.30	4.68E-03
205	B2M	Beta-2-microglobulin	P61769	261	37	2	6.06	13.82	0.105 ± 0.030	0.849 ± 0.082	0.12	2.71E-05
213	SPARC	SPARC	P09486	262	16	4	4.73	35.46	0.395 ± 0.070	1.816 ± 0.320	0.22	2.51E-03
235	ASS1	Argininosuccinate synthase	P00966	213	11	3	8.08	46.78	0.028 ± 0.003	0.163 ± 0.024	0.17	5.52E-04
237	IGFBP2	Insulin-like growth factor-binding protein 2	P18065	366	20	4	7.48	35.87	0.048 ± 0.005	0.184 ± 0.041	0.26	1.06E-02
242	C7	Complement component C7	P10643	455	9	4	6.09	96.65	0.411 ± 0.089	1.231 ± 0.190	0.33	4.53E-03

^*∗*^Ratio of the mean spot intensity of SK-N-SH group divided by SK-N-AS group.

%Cov, the percentage of protein sequence covered by the identified peptides; MW, molecular weight; p*I*, isoelectric point.

**Table 2:** Patient characteristics of 498 pediatric neuroblastomas adopted from the MAQC-III/SEQC study. Data presented as number (%).

	**MAQC-III/SEQC** ^***a***^
	n=498
**Age**	
<18 months	300 (60.2)
≥18 months	198 (39.8)
**Gender**	
Male	287 (57.6)
Female	211 (42.4)
**INSS stage**	
4S	53 (10.6)
1	121 (24.3)
2	78 (15.7)
3	63 (12.7)
4	183 (36.8)
**Risk group**	
High risk	176 (35.3)
**MYCN status**	
Amplification	92 (18.5)
Non-amplification	401 (80.5)
Unknown	5 (1.0)
**HMGB1 mRNA expression** ^***b***^	
Upregulation	55 (11.0)
Downregulation	50 (10.0)
No alteration	393 (79.0)

^*a*^From the NCBI GEO database [28], accession GSE49711 dataset [29].

^*b*^HMGB1 mRNA expression in NB tumors, where upregulation and downregulation were defined by the z-score >1.25 and <-1.25, respectively.

INSS, International Neuroblastoma Staging System.

## Data Availability

The proteomic data and bioinformatic results used to support the findings of this study were included within the Supplementary Information. Clinical and RNA-seq datasets are accessible at the NCBI GEO database [28], accession GSE49711 [29].

## Supplementary Materials

Supplementary Materials Supplementary Table 1: detailed information of protein identities, peptide sequences, and spot intensities of differentially expressed proteins between SK-N-SH and SK-N-AS secretomes. Supplementary Table 2: STRING with GO-term cellular component analysis of the significant proteins. Supplementary Table 3: panther protein classification of the significant proteins. Supplementary Table 4: GO-term biological process of the significant proteins using David functional annotation. Supplementary Table 5: detailed information of patient characteristics, clinical outcomes, and HMGB1 mRNA expression in 498 pediatric neuroblastomas adopted from the NCBI GEO database, accession GSE49711.
